# Increased Circulating MicroRNA-155 as a Potential Biomarker for Breast Cancer Screening: A Meta-Analysis

**DOI:** 10.3390/molecules19056282

**Published:** 2014-05-16

**Authors:** Faliang Wang, Jinchao Hou, Wei Jin, Jiaqiu Li, Yongfang Yue, Hongchuan Jin, Xian Wang

**Affiliations:** 1 Laboratory of Cancer Biology, Institute of Clinical Science, Sir Runrun Shaw Hospital, School of Medicine, Zhejiang University, Hangzhou 310016, China; E-Mails: wangfaliang052@163.com (F.W.); jackie-yue@163.com (Y.Y.); 2 Department of Anesthesiology, the First Affiliated Hospital, School of Medicine, Zhejiang University, Hangzhou 310016, China; E-Mail: houjc_8585@163.com; 3 Department of Clinical Oncology, Sir Runrun Shaw Hospital, School of Medicine, Zhejiang University, Hangzhou 310016, China; E-Mails: 21118179@zju.edu.cn (W.J.); lijiaqiu12@163.com (J.L.); 4 Department of Clinical Oncology, the Children’s Hospital, School of Medicine, Zhejiang University, Hangzhou 310016, China

**Keywords:** breast cancer, circulating miRNAs, biomarker, microRNA-155

## Abstract

The objective of this meta-analysis was to determine the diagnostic accuracy of circulating microRNA-155 (miR-155) for breast cancer (BC). PubMed, Embase, EBSCO (ASP/BSP), Cochrane Library and China National Knowledge Infrastructure (CNKI) were searched up to 30 January 2014 for eligible studies. Quality Assessment of Diagnostic Accuracy Studies (QUADAS) was employed to assess the quality of the included studies. Meta-analysis were performed in Meta-Disc 1.4 and Stata 12.0. Three studies with total 184 BC patients and 75 control individuals were included in this meta-analysis. All of the included studies are of high quality (QUADAS scores 12 or 13). The summary estimates revealed that the pooled sensitivity is 79% (95% confidence interval (CI): 72%–84%) and the specificity is 85% (95% CI: 75%–92%), for the diagnosis of breast cancer. In addition, the area under the summary ROC curve (AUC) is 0.9217. The current evidence suggests that circulating miR-155 has the potential diagnostic value with a high sensitivity and specificity for BC. More prospective studies on the diagnostic value of circulating miR-155 for BC are needed in the future.

## 1. Introduction

Breast cancer (BC) remains the top cancer and the principle cause of death from cancer in women globally, accounting for 28.84% of all cancer cases and 14.49% of cancer-related deaths, respectively [1]. In 2013, an estimated 232,340 new cases of invasive BC and 39,620 BC deaths were expected among American women [1,2]. Since the prognosis of BC is closely related to the extent that how early the disease is diagnosed, efficient and convenient diagnostic methods are urgently needed in the clinical management of BC. Currently, the most efficacious screening tool for BC is mammography. However, it is too expensive to be feasible in most developing countries [3]. Serum-based screening is easier, less invasive and more affordable than mammography. Thus, large part of the efforts focuses on identification of serum-based biomarkers for the diagnosis of BC. At present, the most promising serum markers, CEA and CA15-3, are not sensitive enough for accurate BC diagnosis.

MicroRNAs (miRNAs) are a class of 21–24 nucleotide nonprotein-coding RNAs working as post-transcriptional regulators of gene expression [4,5]. They play important roles in the regulation of target genes by either inducing mRNA degradation or inhibiting translation [6,7]. Numerous studies have shown that aberrant miRNA expression is associated with cancer development and can be detected in serum or plasma and other body fluids [8,9]. There is increasing evidence showing that some circulating miRNAs originated from cancer tissues can be quantitatively measured with established methodology [10,11]. Compared to normal individuals, distinguishable circulating miRNAs expression pattern is observed in BC patients. Therefore, microRNAs in serum and plasma gradually show their advantages in the diagnosis and prognosis prediction of BC [12,13].

MicroRNA-155 (miR-155) is one of the most frequently studied oncomiRNAs. It was reported that miR-155 downregulates SOCS1 in breast cancer, in turn leading to persistent activation of STAT3 signaling and inflammatory cascades. This indicates the communicative role of miR-155 between inflammation and cancer [14]. In breast cancer tissue, the overexpression of miR-155 was observed [15]. Recently, several studies have shown that miR-155 is detected at a high level in the serum of patients with BC [16,17,18,19]. However, the value of miR-155 in the application for BC diagnosis varied in the different studies. Therefore, systematic analysis of these data may be valuable to finally confirm the application potential of miR-155 as a biomarker for BC. The aim of this meta-analysis is to explore the potential value of miR-155 in the diagnosis of BC which, to the best of our knowledge, has not been previously performed.

## 2. Results

**Figure 1:**
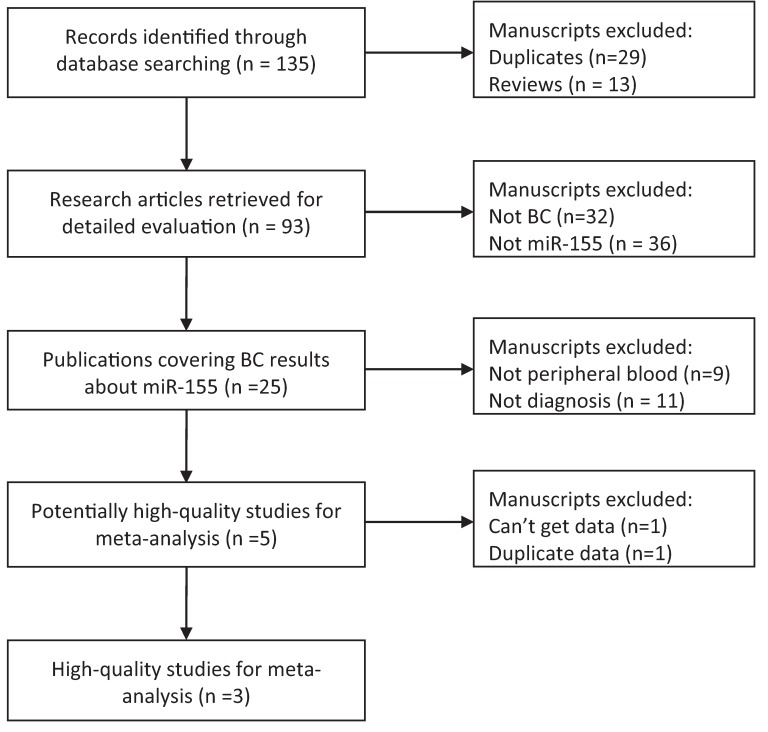
Flow diagram for studies retrieved through the searching and selection processes.

The initial search returned a total of 135 manuscripts, among which 29 duplicated hits and 13 reviews were excluded. The left 93 research articles are subject to the next step evaluation. Thirty two manuscripts were excluded from analysis as the carcinoma was not BC, leaving 61 studies available for further full text review. After carefully reading the text, 36 manuscripts were excluded as other miRNAs rather than miR-155 were focused. Of the remaining 25 manuscripts, samples of nine studies were not from peripheral blood, 11 studies were not diagnostic research, and one study failed to publish detailed information. In the remaining four studies, there were two studies with duplicated data. Thus, the meta-analysis was performed on the final three studies [17,18,20] (Figure 1).

### 2.1. Characteristics of Included Studies

In these eligible articles, all of the 184 BC patients had been histopathologically confirmed. Additionally, all patients were classified by TNM stage. All 75 control individuals were healthy volunteers who had never been diagnosed with a malignant tumor. Therefore, the three studies including 184 patients and 75 control samples reported the quantity of miR-155 in peripheral blood were used in this meta-analysis. All the studies were published from 2012 to 2013. In these researches, miR-155 was detected by reverse transcription Polymerase Chain Reaction (RT-PCR). The fold changes in miR-155 expression were calculated using the 2^−ΔΔCt^ method. These results were summarized in Table 1.

**Table 1:** Summary of studies using miR-155 as a biomarker of BC and study quality assessment.

Authors [ref]	Year	Patients (Control)	QUADAS Scores	Stage I, II%	Mean Age	AUC	Endogenous Control	Cut-off	Sen%	Spe%
Zhao *et al.* [20]	2012	20(10)	12	100	53	0.97	RNU6B	0.95	100	90
Sun *et al.* [17]	2012	103(55)	13	63.1	51	0.801	cel-mir-39	1.911	65.0	81.8
Mar-Aguilar *et al.* [18]	2013	61(10)	12	44.3	53	0.9944	18S RNA	7.92	94.40	100.00

### 2.2. Quality of Included Studies

The three studies were scored by QUADAS by two independent reviewers (F.L.W and J.C.H). The QUADAS analysis showed that all studies got scores of 13 or 12, indicating the high quality of included studies (Table S1).

### 2.3. Description of Outcomes

Sensitivity, specificity, positive LR, negative LR, SROC and DOR were evaluated among the included studies.

### 2.4. Data Analysis

#### 2.4.1. Sensitivity and Specificity

**Figure 2:**
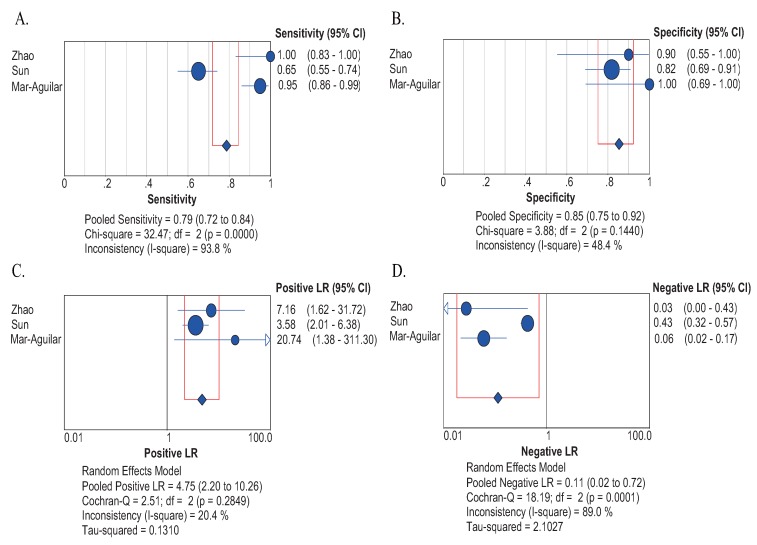
The pooled diagnostic indices for the diagnosis of BC through circulating miR-155. (**A**) The pooled sensitivity for the diagnosis of BC through circulating miR-155; (**B**) the pooled specificity for the diagnosis of BC through circulating miR-155; (**C**) the pooled positive likelihood for the diagnosis of BC through circulating miR-155; (**D**), the pooled negative likelihood ratio for the diagnosis of BC through circulating miR-155.

Considering that a common effect size was shared among studies and significant heterogeneity existed for sensitivity and specificity (I^2^ = 93.8% and 48.4%, respectively), the random effect model was employed. As displayed in Figure 2A,B, the results showed that the pooled sensitivity and specificity were 0.79 (95% CI = 0.72–0.84) and 0.85 (95% CI = 0.75–0.92), respectively.

#### 2.4.2. Positive LR and Negative LR

Since common effect size was shared among studies and significant heterogeneity existed (I^2^ = 20.4% and 89.0%, respectively), random effect model was used. The results revealed that the combined PLR was 4.75 (95% CI = 2.20–10.26), indicating that patients with BC had a nearly 5-fold higher chance of being miR-155 test-positive compared with others without BC (Figure 2C). In respect to NLR, the pooled NLR is 0.11 (95% CI = 0.02–0.72) (Figure 2D).

#### 2.4.3. SROC and DOR

**Figure 3:**
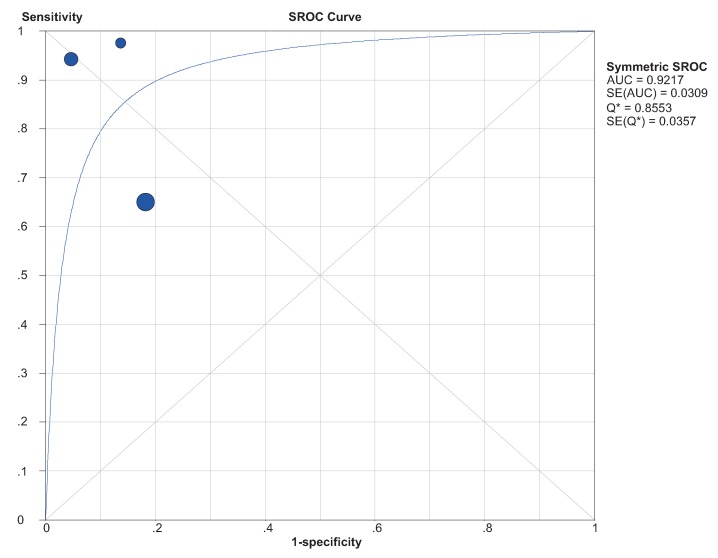
Summary receiver operating characteristics (SROC) curve for the diagnosis of BC through circulating miR-155.

The SROC curve for the included studies was shown in Figure 3. The pooled area under curve (AUC) of SROC was 0.9217 (95% CI = 0.896–0.987) and the DOR is 65.591 (95% CI: 3.669–1172.6).

### 2.5. Publication Bias

**Figure 4:**
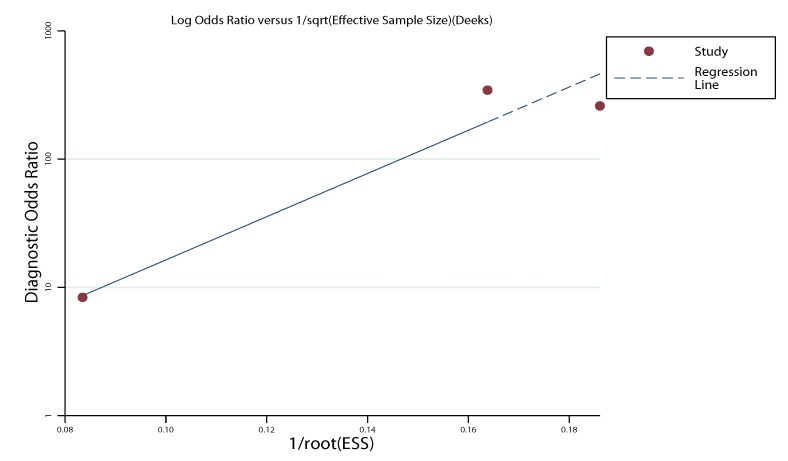
Funnel plot for the assessment of potential bias in miR-155 assays.

To assess the publication bias in this study, funnel plots was used in the meta-analysis. The funnel plot demonstrates a somehow asymmetric curve which can be explained by the limited number of included studies (Figure 4). The *p*-value of the test was 0.124. Therefore, there was no evidence showing that publication bias existed. 

### 2.6. Threshold Effect

The threshold effect is due to differences in the sensitivity and specificity, and Spearman correlation coefficient of sensitivity and specificity is a good approach to evaluate the threshold effect [21]. In this meta-analysis, the Spearman correlation coefficient of sensitivity and 1-specificity was −0.500 with a *p* value of 0.667, suggesting that there is no heterogeneity from threshold effect. The number of patients, the representation of the participants (stage I, II%) and the endogenous control might contribute to the heterogeneity in this meta-analysis.

## 3. Discussion

CEA and/or CA15-3 are currently used as serum markers for BC. Unfortunately, these markers have low sensitivity and specificity for BC screening. MicoRNAs are small non-coding RNA molecules with 21–24 nucleotides in length. Some miRNAs are closely related to the development, invasion and metastasis of tumors [22,23,24,25,26,27]. Calin *et al.* [28] showed that half of the known miRNAs are in cancer-associated genomic regions or fragile sites, suggesting they may be involved in the initiation and progression of human malignancies. MicoRNAs are thus potential markers to detect BC and monitor treatment response of BC patients.

Studies have been showing that miRNAs derived from epithelial tumors are rapidly released into the blood stream [11,29]. More and more attention has been paid to the circulating miRNAs including miR-155 as the early diagnostic biomarker for BC [17,18,19,30]. We conduct this meta-analysis to provide an up-to-date and comprehensive analysis of the feasibility and accuracy of circulating miR-155 for the diagnosis of BC. As far as we know, this is the first meta-analysis about the diagnostic value of miR-155 for BC.

In this meta-analysis, we show that the pooled sensitivity and specificity are 0.79 (95% CI: 0.72–0.84) and 0.85 (95% CI: 0.75–0.92), respectively. Sun *et al.* [17] found that the optimal sensitivity and specificity were 4.9% and 99.1% for CA15-3, 7.8% and 98.2% for CEA, 48.5% and 56.4% for TPS. Thus, circulating miR-155 is more suitable for screening BC compared to conventional serum biomarkers. The diagnostic odds ratio (DOR) defined as the ratio of the odds of a true-positive to the odds of a false-positive, is a single indicator of diagnostic test accuracy that combines the sensitivity and specificity data into a single indicator [31]. The value of DOR ranges from 0 to infinity with higher values indicating better discriminatory test performance. The DOR value of 65.591 indicates that the circulating miR-155 could be a useful biomarker for BC patients’ diagnosis. SROC is usually used to summarize overall test performance, and AUC is calculated to evaluate accuracy of the selected indicator. To demonstrate excellent accuracy, the valve of AUC should be more than 0.97. An AUC of 0.93 to 0.96 is considered to be very good and 0.75 to 0.92 is good. However, a value of less than 0.75 can be still reasonable, while the test will have obvious deficiency in its diagnostic accuracy, approaching a random test [32,33]. In this meta-analysis, we show that circulating miR-155 demonstrates good accuracy in the diagnosis of BC, with an AUC of 0.92. Overall, circulating miR-155 has good sensitivity and specificity in the diagnosis of BC.

Heterogeneity should be analyzed when interpreting the results for meta-analysis. One of the primary causes of heterogeneity in test accuracy studies is threshold effect, which arises when differences in sensitivities and specificities occur due to different cut-offs or thresholds used in different studies to define a positive or negative test result. For different cut-off values were used among the three studies, we used the Spearman correlation coefficient to analyze the threshold. The Spearman correlation coefficient of sensitivity and 1-specificity is −0.500 (*p* = 0.667), indicating that there is no heterogeneity in the threshold. Although the detecting methods for circulating miR-155 are all based on reverse transcriptional PCR (RT-PCR), there are no unified primers and endogenous controls for qPCR analysis. Therefore, different laboratories take different measures to quantify the circulating miR-155, which may contribute to sources of heterogeneity. In addition, the number of patients and the representation of the participants (stage I, II%) in different studies may also involved in forming heterogeneity.

Although we tried to avoid the biases in the meta-analysis process, there are still several limitations to our study. Firstly, the evaluation of circulating miR-155 as a novel marker in BC diagnosis just appeared in recent years, so this meta-analysis only included three studies. Secondly, two of the three included studies have small sample sizes [18,20]. Due to the two points, the study size obtained in this meta-analysis is relatively small. This may result in an unstable result and decrease the significance of this meta-analysis. Thirdly, only one study focus on early-stage breast cancer (stage I and II), while the other two both have advanced-stage breast cancer (stage III and IV). This may undermine the value of circulating miR-155 used for screening early-stage breast cancer. Fourthly, the index test results were not interpreted without knowledge of the results of the reference standard in all the three studies. This may affect the determination of the index test results. Fifthly, North American and northern European countries have the highest incidence rates of BC [34]. However, no relevant studies from these areas could be included in this meta-analysis. Lastly, except for breast cancer, miR-155 is related to haematopoietic lineage differentiation, immunity, inflammation and cardiovascular diseases [35]. So when we apply circulating miR-155 in the screening or diagnosis of breast cancer, the related disease should be considered seriously. In conclusion, despite of the limitations mentioned above, the current evidence suggests that circulating miR-155 has a potential diagnostic value in the diagnosis of BC. Certainly, large-scale prospective studies are needed in the future.

## 4. Materials and Methods

### 4.1. Search Strategy

We carried out a comprehensive search strategy in various databases including PubMed, Embase, EBSCO (ASP/BSP), Cochrane Library and China National Knowledge Infrastructure (CNKI) to seek out the articles studying the association between the level of circulating miR-155 and BC diagnosis up to 30 January 2014. The key words employed for literature retrieval are “microRNA-155” or “miR-155” or “miRNA-155” and “breast cancer” or “breast tumor” or “breast carcinoma” or “breast neoplasm” and “serum” or “sera” or “blood” or “plasma”. To obtain additional relevant articles, we scanned conference summaries and reference lists of articles identified in the initial search and even contacted authors to get additional information if necessary.

### 4.2. Eligibility Criteria

All publications identified by our search strategy were independently assessed by two reviewers (F.L.W and J.C.H). Any disagreement on controversial studies was resolved by fully discussion to consensus. Studies were included if they meet the following inclusion criteria: (1) the diagnosis of BC was made based on histopathological confirmation, which is widely regarded as the gold standard for BC diagnosis; (2) studies detecting miR-155 concentration must be in peripheral blood; (3) peripheral blood must have been collected for miR-155 analysis before any treatment; (4) studies presenting sufficient data to allow construction of two-by-two tables; and (5) patients with benign disease or healthy people served as the control group. Additionally, study exclusion criteria were: (1) duplicate publications; (2) unqualified data; and (3) studies with fewer than 20 patients; All of the literatures in line with the above criteria are considered to be qualified studies.

### 4.3. Data Abstraction

Two reviewers (F.L.W and J.C.H) independently extracted data and reached consensus on all the terms. We recorded the following information about each eligible trial: description of study population (mean age and clinical stage), study details (first author, year of publication and endogenous control), data for two-by-two table (cut-off, sensitivity and specificity) and study design.

### 4.4. Quality Assessment

The quality of each study was scored independently by two reviewers (F.L.W and J.C.H) with the Quality Assessment of Diagnostic Accuracy Studies (QUADAS) tool which features 14 questions and demonstrated to be efficient for the quality assessment of diagnostic accuracy (Table S1) [36]. Each question should be answered with “yes”, “no”, or “unclear”. An answer of “yes” will get one score, while the “no” or “unclear” will gain a score of zero.

### 4.5. Statistical Analysis

Standard methods recommended for diagnostic accuracy meta-analysis were used [37]. The sensitivity and specificity of each study were used to construct a 2 × 2 contingency table, and the patient numbers were used to calculate the overall diagnostic accuracy. The bivariate meta-analysis model was employed to summarize the sensitivity, specificity, positive likelihood ratio (PLR), negative likelihood ratio (NLR), diagnostic odds ratio (DOR) and generate the bivariate summary receiver operator characteristic (SROC) curve [38]. The pooled sensitivity, specificity and other related indexes across studies were calculated using a random-effects model [39]. Chi-square and I^2^ test were used to assess the heterogeneity in studies. A value of *p* less than 0.05 and I^2^ more than 50% indicated the existence of significant heterogeneity [40,41].

The publication bias of selected studies was assessed using linear regression test of funnel plot asymmetry. To detect cut-off threshold effects, the relationship between sensitivity and specificity was evaluated by the Spearman correlation coefficient. All analyses were performed using two statistical software programs: Stata, version 12 (Stata Corporation, College Station, TX, USA) and Meta-Disc 1.4 for Windows (XI Cochrane Colloquium, Barcelona, Spain) [21]. All statistical tests were two sided, and significance was set at *p* < 0.05.

## 5. Conclusions

Our meta-analysis suggests that circulating miR-155 has the potential diagnostic value with a high sensitivity and specificity for BC. More prospective studies on the diagnostic value of circulating miR-155 for BC are needed in the future.

## Supplementary Materials

Supplementary materials can be accessed at: http://www.mdpi.com/1420-3049/19/5/6282/s1.
