# Supplementary material for: Increased Circulating MicroRNA-155 as a Potential Biomarker for Breast Cancer Screening: A Meta-Analysis
Source: Molecules. 2014 May 16;19(5):6282–93. doi: 10.3390/molecules19056282 (PMC6270988; doi:10.3390/molecules19056282)
Supplement: Supplementary file 1 [file molecules-19-06282-s001.pdf]

Supplementary Material

Table S1. The quality assessment of diagnostic accuracy studies (QUADAS).

Item No.	Description	Zhao *et al.*	Sun *et al.*	Mar-Aguilar *et al.*
1	Was the spectrum of patients representative of the patients who will receive the test in practice?	yes	yes	yes
2	Were selections criteria clearly described?	yes	yes	yes
3	Is the reference standard likely to correctly classify the target condition?	yes	yes	yes
4	Is the time period between reference standard and index test short enough to be reasonably sure that the target condition did not change between the two tests?	yes	yes	yes
5	Did the whole sample or a random selection of the sample receive verification using a reference standard of diagnosis?	yes	yes	yes
6	Did patients receive the same reference standard regardless of the index test result?	yes	yes	yes
7	Was the reference standard independent of the index test (*i.e.*, the index test did not form part of the reference standard)?	yes	yes	yes
8	Was the execution of the index test described in sufficient detail to permit replication of the test?	yes	yes	yes
9	Was the execution of the reference standard described in sufficient detail to permit replication of the test?	yes	yes	yes
10	Were the index test results interpreted without knowledge of the results of the reference standard?	no	no	no
11	Were the reference standard results interpreted without knowledge of the results of the index test?	yes	yes	yes
12	Were the same clinical data available when tests results were interpreted as would be available when the test is used in practice?	yes	yes	yes
13	Were uninterpretable/intermediate test results reported?	no	yes	no
14	Were withdrawals from the study explained?	yes	yes	yes
	Total scores	12	13	12

© 2014 by the authors; licensee MDPI, Basel, Switzerland. This article is an open access article distributed under the terms and conditions of the Creative Commons Attribution license (http://creativecommons.org/licenses/by/3.0/).
